# Wall shear stress–related plaque growth of lipid-rich plaques in human coronary arteries: an near-infrared spectroscopy and optical coherence tomography study

**DOI:** 10.1093/cvr/cvac178

**Published:** 2022-12-28

**Authors:** Eline M J Hartman, Giuseppe De Nisco, Annette M Kok, Mariusz Tomaniak, Fay M A Nous, Suze-Anne Korteland, Frank J H Gijsen, Wijnand K den Dekker, Roberto Diletti, Nicolas M D A van Mieghem, Jeroen M Wilschut, Felix Zijlstra, Anton F W van der Steen, Ricardo P J Budde, Joost Daemen, Jolanda J Wentzel

**Affiliations:** Department of Cardiology, Erasmus MC, Dr. Molewaterplein 40, P.O. Box 2040, 3000 CA Rotterdam, The Netherlands; PoliToBIOMed Lab, Department of Mechanical and Aerospace Engineering, Politecnico di Torino, 10129 Torino, Italy; Department of Cardiology, Erasmus MC, Dr. Molewaterplein 40, P.O. Box 2040, 3000 CA Rotterdam, The Netherlands; Department of Cardiology, Erasmus MC, Dr. Molewaterplein 40, P.O. Box 2040, 3000 CA Rotterdam, The Netherlands; First Department of Cardiology, Medical University of Warsaw, 02-091 Warsaw, Poland; Department of Cardiology, Erasmus MC, Dr. Molewaterplein 40, P.O. Box 2040, 3000 CA Rotterdam, The Netherlands; Department of Radiology and Nuclear Medicine, ErasmusMC, 3000 CA Rotterdam, The Netherlands; Department of Cardiology, Erasmus MC, Dr. Molewaterplein 40, P.O. Box 2040, 3000 CA Rotterdam, The Netherlands; Department of Cardiology, Erasmus MC, Dr. Molewaterplein 40, P.O. Box 2040, 3000 CA Rotterdam, The Netherlands; Department of Cardiology, Erasmus MC, Dr. Molewaterplein 40, P.O. Box 2040, 3000 CA Rotterdam, The Netherlands; Department of Cardiology, Erasmus MC, Dr. Molewaterplein 40, P.O. Box 2040, 3000 CA Rotterdam, The Netherlands; Department of Cardiology, Erasmus MC, Dr. Molewaterplein 40, P.O. Box 2040, 3000 CA Rotterdam, The Netherlands; Department of Cardiology, Erasmus MC, Dr. Molewaterplein 40, P.O. Box 2040, 3000 CA Rotterdam, The Netherlands; Department of Cardiology, Erasmus MC, Dr. Molewaterplein 40, P.O. Box 2040, 3000 CA Rotterdam, The Netherlands; Department of Cardiology, Erasmus MC, Dr. Molewaterplein 40, P.O. Box 2040, 3000 CA Rotterdam, The Netherlands; Department of Radiology and Nuclear Medicine, ErasmusMC, 3000 CA Rotterdam, The Netherlands; Department of Cardiology, Erasmus MC, Dr. Molewaterplein 40, P.O. Box 2040, 3000 CA Rotterdam, The Netherlands; Department of Cardiology, Erasmus MC, Dr. Molewaterplein 40, P.O. Box 2040, 3000 CA Rotterdam, The Netherlands

**Keywords:** Coronary artery disease, Near-infrared spectroscopy, Intravascular ultrasound, Optical coherence tomography, Wall shear stress

## Abstract

**Aims:**

Low wall shear stress (WSS) is acknowledged to play a role in plaque development through its influence on local endothelial function. Also, lipid-rich plaques (LRPs) are associated with endothelial dysfunction. However, little is known about the interplay between WSS and the presence of lipids with respect to plaque progression. Therefore, we aimed to study the differences in WSS-related plaque progression between LRPs, non-LRPs, or plaque-free regions in human coronary arteries.

**Methods and results:**

In the present single-centre, prospective study, 40 patients who presented with an acute coronary syndrome successfully underwent near-infrared spectroscopy intravascular ultrasound (NIRS-IVUS) and optical coherence tomography (OCT) of at least one non-culprit vessel at baseline and completed a 1-year follow-up. WSS was computed applying computational fluid dynamics to a three-dimensional reconstruction of the coronary artery based on the fusion of the IVUS-segmented lumen with a CT-derived centreline, using invasive flow measurements as boundary conditions. For data analysis, each artery was divided into 1.5 mm/45° sectors. Plaque growth based on IVUS-derived percentage atheroma volume change was compared between LRPs, non-LRPs, and plaque-free wall segments, as assessed by both OCT and NIRS. Both NIRS- and OCT-detected lipid-rich sectors showed a significantly higher plaque progression than non-LRPs or plaque-free regions. Exposure to low WSS was associated with a higher plaque progression than exposure to mid or high WSS, even in the regions classified as a plaque-free wall. Furthermore, low WSS and the presence of lipids had a synergistic effect on plaque growth, resulting in the highest plaque progression in lipid-rich regions exposed to low shear stress.

**Conclusion:**

This study demonstrates that NIRS- and OCT-detected lipid-rich regions exposed to low WSS are subject to enhanced plaque growth over a 1-year follow-up. The presence of lipids and low WSS proves to have a synergistic effect on plaque growth.

## Graphical Abstract

**Graphical Abstract:**
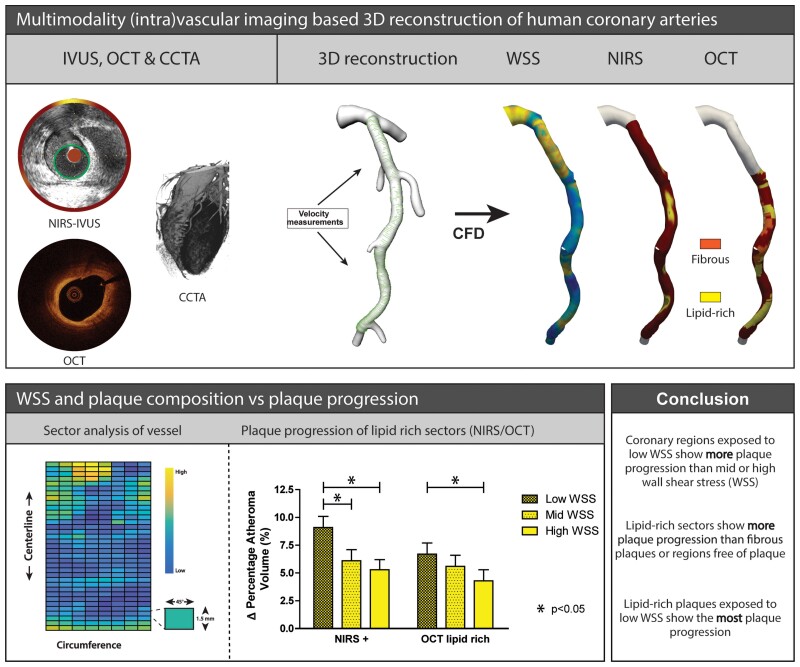

**Translational perspective**

The development and location of atherosclerosis is known to be a multifactorial process. Many factors are individually associated with the development of atherosclerosis, such as wall shear stress (WSS) and lipid-rich plaque (LRPs). In this study, we show that there is a synergistic effect between low WSS and LRPs on plaque progression. From a translational perspective, regions with endothelial dysfunction due to simultaneous exposure to inflammation, which is caused by local lipid infiltration, and to low WSS, are most susceptible to plaque progression. Future studies are needed to demonstrate whether these LRPs exposed to low WSS showing plaque progression are related to future cardiovascular events.

## 1. Introduction

Coronary atherosclerosis is a lipid-driven inflammatory disease and the most common cause of ischaemic heart disease.^1^ In the last few decades, it has been well established that blood flow–induced wall shear stress (WSS) plays a crucial role in the process of plaque initiation, because low WSS is responsible for local regions of endothelial dysfunction.^2–4^ In these regions, lipids infiltrate the vessel wall, eventually resulting in atherosclerotic plaques.^5^ In turn, lipids inside the plaque have been proved to influence local inflammation and thereby stimulate further plaque progression.^6^ Interestingly, lipids in the vessel wall as detected by near-infrared spectroscopy (NIRS) have also been related to endothelial dysfunction in the coronary arteries, independent of the plaque burden.^7^ Despite expanding knowledge on the individual roles of WSS and plaque composition (e.g. lipid-rich) on plaque growth, little is known about their interplay and the combined effect on plaque progression. The effect that low WSS has on plaque progression via endothelial dysfunction might be enhanced by an inflamed environment of lipid-rich plaques (LRPs) and the related endothelial dysfunction. We hypothesize that regions with endothelial dysfunction due to both low WSS and inflammation, which is caused by local lipid infiltration, are most susceptible to plaque progression. The latter could be of particular interest since progression has been demonstrated to precede plaque rupture and subsequent events.^8^

At present, several intravascular imaging modalities are available to visualize these LRPs *in vivo*.^9,10^ Currently, there are several devices available in the clinic that can visualize plaque volume and/or plaque components. Among them, optical coherence tomography (OCT) is a high-resolution imaging technique able to visualize different layers of a healthy vessel wall and lipids within the vessel wall. However, it lacks enough penetration depth to assess plaque volumes.^11^ The introduction of dual-sensor NIRS intravascular ultrasound (NIRS-IVUS) allowed simultaneous assessment and thus a co-localization of lipids and plaque volume.^12^ By combining these intravascular imaging techniques with computational fluid dynamics (CFD) to compute WSS, the interplay between WSS and LRP can be investigated.

The present study uses serial multimodality imaging, three-dimensional (3D)-vessel reconstruction, and CFD to investigate the interaction between WSS and intra-plaque lipids on plaque progression in human coronary arteries.

## 2. Methods

### 2.1 Patient inclusion

The IMPACT study was a prospective, single-centre, serial multimodality imaging study designed to evaluate the association between biomechanical parameters and the natural history of atherosclerotic disease in non-stented coronary arteries. Haemodynamically stable patients with an acute coronary syndrome (ACS), who had at least one non-stented non-culprit coronary vessel accessible with intracoronary imaging catheters and a Doppler velocity wire, were eligible for enrolment. Patients were treated according to the clinical guidelines and discharged on dual-antiplatelet therapy and high-intensity lipid-lowering therapy (rosuvastatin ≥20 mg, atorvastatin ≥40 mg, or the PCSK9-inhibitor). The most important exclusion criteria were previous coronary artery bypass graft surgery, three-vessel disease, impaired renal function (creatinine clearing <50 mL/min), left ventricular ejection fraction <30%, and atrial fibrillation. Written informed consent was obtained from all patients. The study protocol was approved by the local medical ethics committee of the Erasmus MC (MED2015-535, NL54519.078.15) and registered (ISCRTN: 43170100). The IMPACT study was conducted in accordance with the World Medical Association Declaration of Helsinki (64th WMA General Assembly, Fortaleza Brazil, October 2013) and Medical Research involving the human subject act (WMO).

### 2.2 Clinical data acquisition

After successful percutaneous coronary intervention (PCI) of the culprit lesions, a non-culprit artery segment with a minimum of 30 mm of length and two readily identifiable side branches (diameter >1.5 mm) was selected as the study segment. An NIRS-IVUS catheter (TVC Insight Coronary Imaging Catheter, InfraRedX, Burlington, MA, USA) was advanced and positioned distally from the distal side branch. An automated pullback (0.5 mm/s) was performed. Subsequently, an OCT catheter (Dragonfly Optis Imaging Catheter, St Jude Medical, St Paul, MN, USA) was positioned at the same anatomical location as the NIRS-IVUS catheter, and an automated pullback (36 mm/s) was performed. Finally, invasive local flow measurements were performed using a ComboWire (Phillips Volcano, Zaventem, Belgium) at different locations between the side branches throughout the study segment to assess the flow distribution. This invasive imaging protocol was repeated at a 1-year follow-up. In addition, 1 month after the index procedure, the patients visited the outpatient clinic to undergo a coronary computed tomography angiogram (CCTA) according to a standard prospectively electrocardiogram-triggered clinical protocol [SOMATOM Force (3rd generation dual-source CT scanner), Siemens Healthineers, Germany].

### 2.3 Invasive imaging analysis

Data were anonymized and analysed offline. Analyses of both IVUS and OCT data were performed using QCU-CMS software (version 4.69, LKEB, Division of image processing, LUMC, Leiden, The Netherlands) blinded for the other invasive imaging. IVUS images were gated using an in-house developed MATLAB (V.2017B, Mathworks Inc., USA) algorithm, generating one IVUS frame in every end-diastolic phase of the cardiac cycle to correct for variations in lumen and vessel size due to cardiac contraction. Lumen and external elastic membrane (EEM) contours were semi-automatically delineated and segmented. An intra-observer analysis was performed in a random sample of 5 IVUS pullbacks (748 frames) with at least a 2-month interval. A good reproducibility of the EEM area, lumen area, and plaque area was found [0.996 (95% confidence interval, CI 0.996–0.997), 0.983 (95% CI 0.963–0.990), and 0.958 (95% CI 0.939–0.970)]. In the IVUS frames, calcium was identified as a bright signal with echo lucent shadow. Calcium angles were segmented with the protractor in the centre of the lumen. For each degree in an NIRS-IVUS frame, the NIRS signal was analysed and was NIRS-positive if the signal had a high probability (>0.6) for the presence of lipids. The Lipid Core Burden Index (LCBI) (area positive for NIRS signal/total area × 100) was calculated for each vessel. Regions with the highest lipid content per 4 mm (maxLCBI4mm) were identified for vessel characterization.^13,14^ OCT images were analysed every millimetre (one out of five frames) according to the consensus standard.^11^ Lumen contours were segmented, and plaque components visible on OCT were manually segmented by drawing angles from the centre of the lumen. In detail, a LRP was defined as an inhomogeneous signal on OCT, with a drop in attenuation and no visible EEM. A lipid pool was identified in the case of an overlying signal-rich cap structure with a sudden drop of the signal and classified as a fibrous cap atheroma (FCA). A fibrous plaque was defined as a relatively homogeneous signal on the OCT and identifiable EEM (intima-media thickness >0.5 mm). A plaque-free wall was defined as a healthy wall with a visible three-layered structure with an intima-media thickness <0.5 mm.

### 2.4 3D-reconstruction and CFD

**Figure 1:**
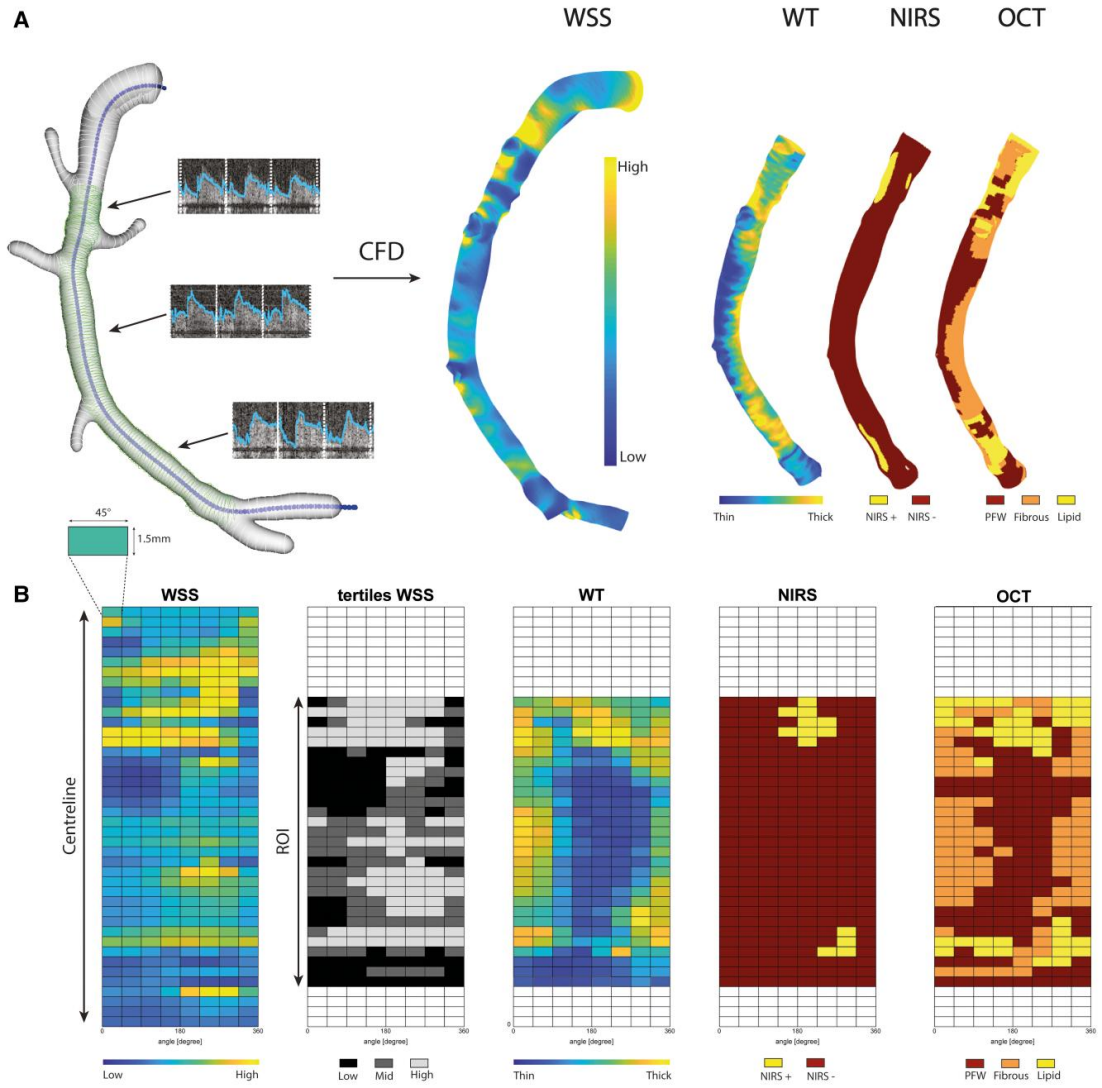
Methodology overview of 3D reconstruction, WSS, and data analysis matching plaque components. (*A*) Example of a 3D-reconstructed RCA. IVUS-derived lumen contours (green) were fused with the CT-derived vessel centreline (blue) and CT-derived side branch contours (white). By adding local flow measurements and using CFD, WSS was calculated in these reconstructed models. NIRS- and OCT-derived plaque phenotypes were matched and plotted on the IVUS-derived 3D reconstruction, the ROI. (*B*) From the 3D-reconstructed vessels, 2D maps were created by cutting the vessel open in the longitudinal direction. For statistical analysis, the 2D maps were divided into sectors of 1.5 mm/45°. Examples of 2D maps in this overview are as follows: the continuous WSS, the WSS tertiles (low, mid, high), wall thickness (WT), NIRS-derived plaque phenotype, and OCT-derived phenotype.

By fusing the 3D spatial information of the coronary vessel centreline segmented from the CCTA and the lumen contours extracted from the IVUS, a 3D reconstruction was made in MeVisLab (MeVis Medical Solutions AG, Bremen, Germany). The two imaging modalities were matched using large side branches as landmarks, both in the longitudinal and in the rotational directions. For subsequent CFD, reliable inlet and outlets were needed. Therefore, the regions proximal and distal to the IVUS-derived region of interest (ROI), as well as side branches (>1.5 mm), were segmented on the CCTA and scaled and fused with the 3D reconstruction^15,16^ (*Figure 1A*). For the final analysis, only the IVUS-derived ROI was considered. CFD analyses were used to obtain the local WSS according to a previously described methodology.^17^ In brief, a time-dependent CFD simulation was performed in each reconstructed 3D geometry, assuming blood as an incompressible, non-Newtonian fluid, and modelled using the Carreau fluid model (Fluent, v.17.1, ANSYS Inc.). The vessel lumen was considered rigid and subjected to a no-slip condition. For the CFD simulations, in- and outflow boundary conditions were derived from intravascular Doppler measurements. The quality was examined in a consensus meeting of experts (A.M.K., E.M.J.H., F.J.H.G., and J.J.W.) based on the flow signal’s quality, repeatability, and consistency. For the inflow boundary condition of the time-dependent CFD simulation, a flat velocity profile was applied with a time-dependent waveform derived from the most proximal flow measurement of good quality. Furthermore, for the outflow boundary conditions, the flow distribution through the side branches was calculated based on the intravascular flow measurements at different locations in the coronary artery. A previously described scaling law was used for regions with no reliable flow measurements to determine the flow ratio between the main and the side branches.^18^

### 2.5 Data analysis

By using side branches as landmarks, both the analysed OCT data and the segmented IVUS follow-up contours were matched to the IVUS baseline pullback. Matching was performed in both longitudinal and circumferential directions. All matched and analysed data were mapped and interpolated on the IVUS-based ROI on the 3D-mesh geometry using VMTK (Orobix, Bergamo, Italy) and MATLAB (v2017b, Mathworks Inc., Natick, MA, USA) (*Figure 1A*). For further analyses, the 3D ROIs were converted into a two-dimensional (2D) map by folding the vessel open in a longitudinal direction (*Figure 1B*). The arteries were split into 1.5 mm segments and divided into 45° sectors (*Figure 1B*). All continuous data were averaged per sector. Plaque was expressed as per cent atheroma volume (PAV) (plaque area volume/total vessel area volume × 100). For each vessel, the cardiac-cycle averaged WSS was divided into vessel-specific tertiles (low, mid, and high), assigning one-third of the sectors in the vessel as exposed to low, mid, or high WSS. Furthermore, since calcium hampers visualization of the EEM, in calcium positive locations, any wall thickness and therefore PAV estimation was not possible. All sectors containing an IVUS-derived calcium angle of more than 90° were excluded from all PAV analyses.^19^ A sector was considered NIRS-positive when at least 50% of a sector was NIRS-positive (>0.6 probability of the presence of lipids). NIRS data were divided into three groups: (i) NIRS-positive sectors, (ii) NIRS-negative plaque sectors (wall thickness >0.5 mm), and (iii) NIRS-negative plaque-free wall sectors (wall thickness <0.5 mm), that is, an NIRS-IVUS-based plaque-free vessel wall (IVUS-PFW). For the OCT-derived plaque classification, a sector was assigned to a group when at least 50% of that sector consisted of an OCT-derived vessel wall feature (FCA, lipid-rich, fibrous, PFW). Since FCA (28/7851 sectors) were scarce, no dedicated statistical analysis was performed, and the sectors classified as FCA were pooled together with the sectors classified as lipid-rich. As such, based on OCT imaging, all sectors were stratified using three categories: (i) LRPs, (ii) fibrous plaques, and (iii) plaque-free wall (OCT-PFW).

### 2.6 Statistical analysis

Normally distributed data were presented as mean standard deviation, and significance was tested using Student’s *t*-test or ANOVA. Non-normally distributed data were reported as medians (inter-quartile range), and the statistical difference was determined by using the Mann–Whitney *U* test.

A statistical analysis of plaque burden changes (follow-up baseline) was performed using linear mixed models with WSS tertiles (low, mid, high) and OCT and NIRS plaque phenotypes of the sectors as fixed factors. The individual vessel was added to the model as a random factor using an unstructured covariance and correlation matrix to take random effects and correlations of the sectors within the individual vessels into account. Since high-intensity lipid-lowering therapy has shown to impact plaque progression and when considering the low compliance of high-intensity lipid-lowering therapy in daily practice,^20–22^ we compared the PAV of patients who remained treated with high-intensity lowering therapy after 1 year with patients on low- or moderate-intensity lipid-lowering therapy. All statistical tests for PAV change were adjusted for baseline PAV since plaques with different phenotypes might present with different plaque sizes at baseline. All the results on plaque progression presented in this study are estimated means of the delta PAV, and a 95% CI. *P* < 0.05 was considered statistically significant.

## 3. Results

Between March 2016 and March 2018, a total of 53 patients were enrolled in the study. Four patients withdrew consent at the 1-month follow-up, and eight patients withdrew at the 1-year invasive imaging procedure. In one patient, there was no possibility of matching between computed tomography and invasive imaging data. As a result, a total of 40 patients with ACS underwent NIRS-IVUS and OCT assessment of a non-culprit coronary artery after successful treatment of the culprit lesion both at baseline and at a 12-month follow-up, and after excluding one patient due to insufficient quality NIRS data and two patients due to a non-analysable OCT. A complete OCT, serial NIRS-IVUS, and CCTA dataset was available on 37 patients (38 vessels). Consequently, 3D lumen geometries for CFD analyses were created and intravascular imaging data were analysed and matched for 38 vessels. The patient characteristics are given in *Table 1*. The mean age was 60 ± 8.8, 92% were male, 46% of the patients were statin naïve at the time of enrolment, and 51% were on high-intensity lipid-lowering therapy at 1-year follow-up.

**Table 1:** Baseline characteristics

Clinical characteristics	*N* = 37 patients
Age, years	60 ± 8.9
Men, *n* (%)	34 (92%)
Body mass index	27 ± 5.0
Diabetes mellitus, *n* (%)	8 (22%)
Hypertension, *n* (%)	9 (24%)
Dyslipidaemia, *n* (%)	16 (42%)
Current smoking, *n* (%)	8 (22%)
Positive family history, *n* (%)	13 (35%)
Previous MI, *n* (%)	8 (22%)
Previous PCI, *n* (%)	11 (30%)
LDL (mmol/L)	2.7 ± 0.9
**Imaged study vessel**	***N* = 38 vessels**
LAD, *n* (%)	14 (36%)
LCX, *n* (%)	9 (23%)
RCA, *n* (%)	16 (41%)
maxLCBI4mm	275 (167–383)

### 3.1 Baseline imaging characteristics

The studied non-culprit segment was located in the left anterior descending in 13 vessels, the left circumflex in 10 vessels, and the right coronary artery (RCA) in 15 vessels. The median length of the IVUS-based ROI was 54 (39–61.5) mm, the time-averaged WSS was 1.10 (0.77–1.73) Pa, and the maxLCBI4mm was 275 (167–338). Exclusion of regions with side branches and dividing the vessels in 1.5 mm by 45° resulted in 9906 sectors. The mean WSS per vessel for the low WSS tertile was 0.47 (0.37–0.69) Pa, for the mid-WSS tertile, it was 0.87 (0.68–1.23) Pa, and for the high WSS tertile, it was 2.04 (1.22–2.85) Pa.

Calcifications >90° were found in 739 sectors at baseline and/or follow-up, and these sectors were excluded from all analyses. Due to guidewire artefacts in the detection of NIRS, 306 sectors at baseline were excluded for the relevant analyses and sub-analyses. In total, 2041 sectors were excluded from the OCT analysis due to problems related to matching, or poor imaging quality (*N* = 1899), or the heterogeneity of plaque composition (*N* = 142). In total, 504 sectors were identified as NIRS-positive, 2667 sectors as NIRS-negative plaque, and 5690 sectors as IVUS-PFW. When using OCT for plaque characterization, 1624 sectors were classified as lipid-rich, 1214 sectors were classified as fibrous, and 4288 sectors were classified as OCT-PFW.

### 3.2 Plaque progression

Firstly, no significant differences were observed in terms of PAV change between patients on high-intensity lipid lowering therapy and those who were either intolerant or on low-intensity lipid therapy after 1-year follow-up (high: 2.7% 95% CI 0.0–5.4, low/non 2.7% 95% CI −0.09–6.3). Consequently, no sub-analysis of the use of high-lipid lowering therapy was performed.

**Figure 2:**
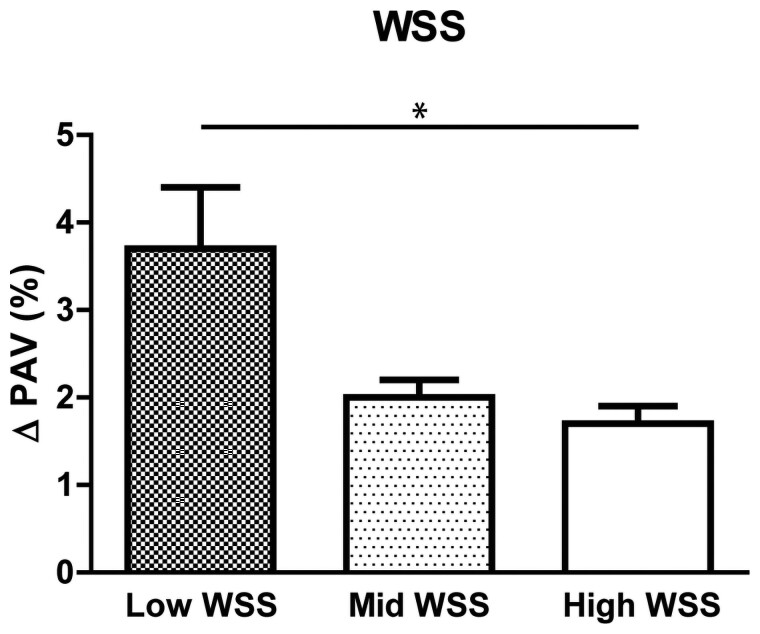
Plaque progression in different WSS tertiles. Change in plaque expressed as Δ PAV for low, mid, and high WSS (*P < 0.05, Estimated mean + Standard Error, linear mixed model).

Secondly, sectors exposed to low WSS demonstrated a significantly higher plaque progression compared with sectors exposed to high WSS (low: 3.7% 95% CI 2.3–5.1, mid: 2.0% 95% CI 1.7–2.4, high: 1.7% 95% CI 1.4–2.1, *P* < 0.001). No differences in plaque progression were found when sectors exposed to mid and high WSS (*P* = 0.73) (*Figure 2*) were compared.

**Figure 3:**
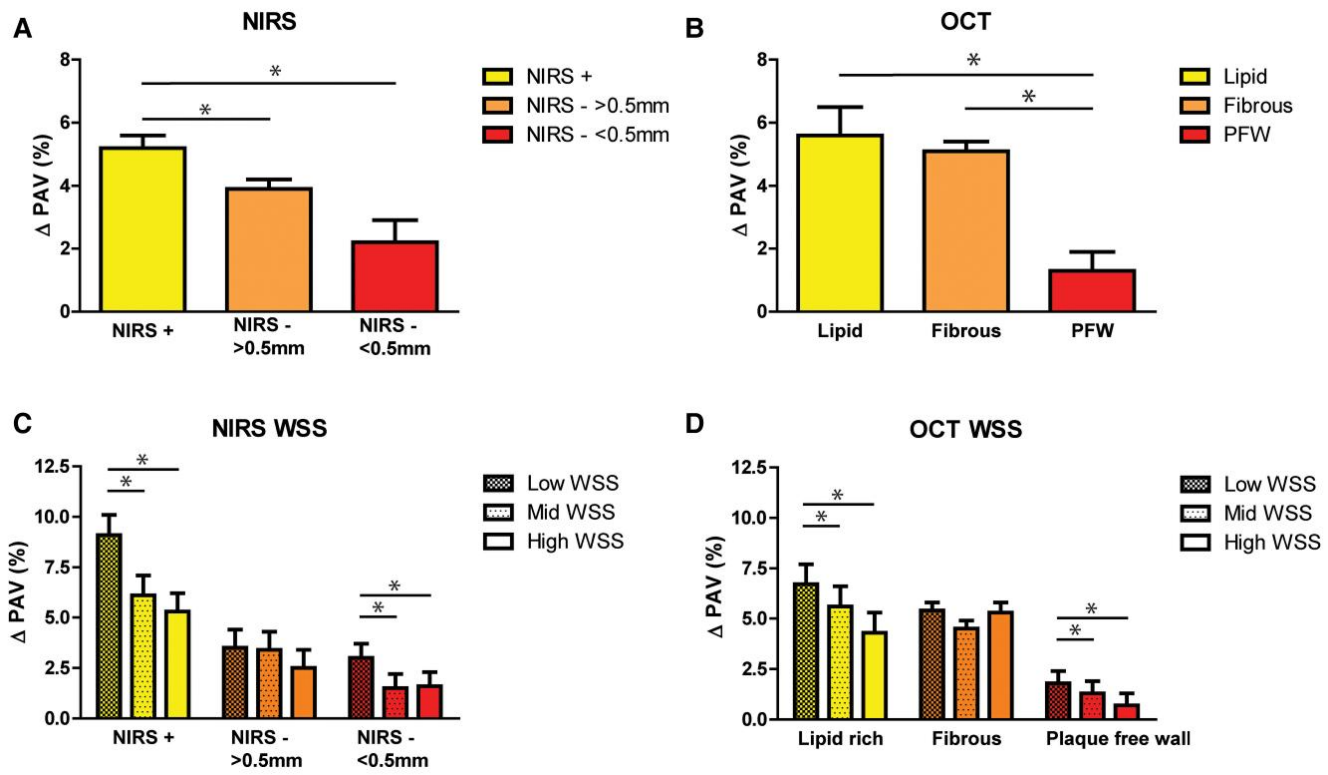
Plaque progression for different plaque phenotypes and in different WSS tertiles. (*A*) Plaque progression for an NIRS-IVUS-derived plaque phenotype LRP (NIRS +), a non-LRP (NIRS - & plaque thickness > 0.5 mm), and an IVUS-based plaque-free wall (NIRS - & plaque thickness < 0.5 mm). (*P < 0.05, Estimated mean + Standard Error, linear mixed model). (*B*) Plaque progression for an OCT-derived plaque phenotype LRP (Lipid), fibrous, and an OCT-based plaque-free wall (PFW). (*P < 0.05, Estimated mean + Standard Error, linear mixed model). Plaque progression for NIRS-IVUS (*C*)- and OCT (*D*)-derived plaque phenotypes divided into low (no pattern), mid (light pattern), and high (dense pattern) WSS. (*P < 0.05, Estimated mean + Standard Error, linear mixed model).

Thirdly, when analysing the effect of NIRS-detected local lipids on plaque progression, lipid-rich sectors showed a significantly higher plaque progression compared with sectors in which lipids were not present (non-LRPs) (LRP: 5.2% 95% CI 4.3–6.0 vs. non-LRP: 3.9% 95% CI 3.3–4.4, *P* = 0.010). Furthermore, LRPs showed more plaque progression than PFW sectors (PFW: 2.2% 95% CI:0.9–3.5%, *P* = 0.001) (*Figure 3A*).

Fourthly, when assessing the differences in plaque progression between sectors that demonstrated various OCT-based plaque components, both fibrous and lipid-rich sectors showed a significantly more pronounced plaque progression than the OCT-based PFW sectors. However, no significant differences in plaque progression were found between lipid-rich and fibrous sectors [Lipid: 5.6% (95% CI: 3.8–7.4), Fibrous: 5.1% (95% CI: 4.6–5.6), PFW: 1.3% (95% CI: 0.1–2.4), *P* < 0.001] (*Figure 3B*).

Finally, lipid-rich sectors exposed to low WSS showed a significantly higher plaque progression compared with those exposed to high WSS, both for OCT-based and NIRS-based LRPs. In the PFW regions (wall thickness <0.5 mm), plaque progression was primarily found in sectors exposed to low WSS and had a significantly higher plaque progression compared with PFW sectors exposed to high WSS. (*Figure 3C and D*). In the linear mixed models, the presence of lipids based on NIRS or OCT and WSS showed a significant interaction effect (*P* < 0.001), indicating that low WSS had a larger effect on plaque progression in the lipid-rich sectors compared with the non-lipid-rich sectors.

## 4. Discussion

In this study, we combined serial multimodality imaging with CFD to investigate the influence and interplay of WSS and the presence of NIRS- and OCT-detected lipids on IVUS-based plaque progression over a 1-year follow-up period in a dedicated single-centre study. We used a detailed sector-based analysis of NIRS-IVUS-derived 3D-CFD models, thereby considering the local heterogeneity in WSS, lipid presence, and plaque progression. The most important findings were: (i) sectors exposed to low WSS displayed more plaque progression than those exposed to mid and high WSS. (ii) In NIRS-detected lipid-rich sectors, more plaque progression was observed than in non-LRP sectors and PFW sectors. (iii) Both lipid-rich and fibrous plaques, as detected by OCT, showed a significantly higher plaque growth compared with PFW sectors. (iv) When combining WSS and plaque components, lipid-rich sectors exposed to low WSS showed the highest plaque progression. (v) In PFW sectors, as determined by either NIRS-IVUS or OCT, plaque progression was the highest in sectors exposed to low WSS.

### 4.1 Plaque progression was most visible in LRPs detected by NIRS

The present study is the first to show that lipid-rich regions, as detected by NIRS, are prone to plaque progression at a 1-year follow-up compared with sectors that do not contain lipids. Our findings correspond well with the earlier studies showing that lipids cause a pro-inflammatory response inside the vessel wall, leading to the influx of inflammatory cells.^5^ Subsequently, the presence of lipids and inflammatory cells stimulates a complex cascade of pathways, finally resulting in plaque progression.^5,6^ Earlier, a pre-clinical histological work demonstrated that NIRS-positive plaques display greater necrotic cores and more inflammation than NIRS-negative plaques, supporting the hypothesis of enhancing plaque growth in lipid-rich regions.^23^

In the LRP study, which was a natural history study, non-culprit vessels with a high lipid content (maxLCBI4mm >400) were at three times higher risk of developing major cardiovascular events than non-culprit vessels with low lipid content.^24^ Besides inflammation, plaque progression has also been demonstrated to be one of the precursors of plaque rupture and subsequent events.^8^ Therefore, our findings might contribute to understanding the evolution of an event-causing lesion.

It is noteworthy that the analyses stratified according to the OCT-detected plaque phenotypes revealed no significant difference in plaque progression between lipid and fibrous sectors. One of the reasons why differences between lipid and fibrous sectors were not seen in the OCT-lipid analysis could be the differences in the detection and analysis of lipids between the two image modalities. Both NIRS and OCT use a near-infrared light-based imaging technique; however, a number of differences have to be addressed. First, in this population, OCT-derived FCAs, with a potentially more vulnerable phenotype, were rare, and thus the total number was to low to perform statistical analyses with FCA as a separate phenotype. Therefore, FCA and all other LRPs were pooled for the analysis, with a caveat of decreased specificity. Second, OCT-detected lipids were based on an expert observer analysis. Conversely, in the NIRS analysis, the presence of lipids was based on an automated analysis of the optical absorption spectrum of different plaque components based on the validation algorithm.^25^ The discrepancy found between OCT and NIRS on lipid detection has been described previously. Previous work demonstrated a modest agreement between NIRS and OCT for lipid detection, partly caused by a 20% false-positive rate of OCT-detected lipid compared with NIRS.^26^ These data suggest that OCT is more sensitive for lipid detection but less specific for the more vulnerable plaque than NIRS imaging.

### 4.2 WSS, plaque composition, and plaque progression

Low WSS has been primarily associated with the initiation process of atherosclerosis, resulting from its detrimental effects on endothelial function, promoting increased permeability of the endothelial cells. The subsequent influx of low-density lipoprotein (LDL) into the vessel wall leads to plaque development.^27^ In this study, we investigated plaque progression using IVUS and found enhanced plaque growth in low WSS regions, in line with that of previous studies.^28,29^ We found that LRP exposed to *low* WSS displayed the highest plaque progression, and we even showed that low WSS and the presence of lipids had a synergistic effect on plaque progression. Both LRPs and low WSS are independently associated with endothelial dysfunction.^3,7^ From a pathophysiological point of view, we hypothesize that the synergistic effect might be related to the combined effect and interaction of low WSS and LRPs on endothelial dysfunction, leading to the highest plaque progression at these lipid-rich locations exposed to low WSS.

At present, little is known about the interplay between LRP and WSS regarding plaque progression. To date, only one study combined serial NIRS-IVUS imaging with WSS and showed that segments with proven *lipid* progression were exposed to *higher* WSS at baseline compared with segments with no lipid progression.^30^ In contrast, the present study focused on the role of WSS in plaque volume progression rather than the change in lipid content. Altogether, these results might implicate different roles of WSS for volumetric plaque progression and plaque vulnerability. This dual role of WSS was recognized previously by Slager *et al*.,^31^ who envisioned that regions exposed to low WSS were more susceptible to plaque initiation and further plaque progression, while the exposure of plaques to high WSS might affect plaque vulnerability.^31^ Although in a previous study we found that LRPs were more often exposed to high WSS,^15^ there were still lipid-rich regions exposed to low WSS^15^ that showed the highest plaque progression. Previous research showed an association between high-risk plaque characteristics being exposed to low WSS at the index procedure with future events after a 3-year follow-up in non-culprit vessels.^32^

Recent research employing multimodality imaging with both OCT and IVUS showed that low WSS and baseline plaque burden could predict disease progression.^33,34^ However, those studies did not find a difference in WSS-related plaque growth among various plaque phenotypes. The reason for these different results compared with our data could be the level of detail that was used in the analysis. Bourantas *et al*. used 3 mm sectors in which the minimum predominant WSS, defined as the 90° arc with the minimum averaged WSS, was selected as a representative for the whole segment. Furthermore, each segment’s plaque phenotype was defined according to the most vulnerable plaque type located in the segment. To investigate the more local effect of WSS and plaque composition and their interaction in order to unravel the underlying mechanisms, we took this one step further and analysed every 1.5 mm/45° sector for WSS and plaque composition. One risk of this relatively high sampling rate is the potential interaction effect between sectors within a vessel. Therefore, we used an advanced statistical random-effects model with an unstructured covariance and correlation matrix to take any correlation effects into account. By using this detailed analysis, we did find a synergistic effect of WSS and the presence of lipids with respect to plaque progression. These data suggest that at a very local level, WSS and the underlying plaque composition interact with each other in their influence on endothelial dysfunction and thus on the pathophysiology leading to plaque growth.

Furthermore, in contrast to our study, lipid tissue in superficial plaque detected by OCT was not identified as a predictor for disease progression. It is plausible that the difference in the definition of LRPs vs. lipid tissue in the superficial plaque (<0.5 mm depth) underlies this discrepancy. Similar to our study, the number of segments with lipid tissue in the superficial plaque in this study was low. For both NIRS-negative plaques (>0.5 mm) and OCT-detected fibrous plaques, no effect of WSS was visible on plaque progression. Of note, both thin (<0.5 mm) NIRS-negative sectors, that is an NIRS-based plaque-free wall, as well as plaque-free wall sectors detected by OCT, showed significant plaque progression in the sectors exposed to low WSS, which is an indirect confirmation of the role of low WSS specifically in early plaque initiation. Irrespective of these findings, key differences in the method of WSS computations should be mentioned. As such, in contrast to the vast majority of other studies, we included side branches in our computational model, which profoundly affects the WSS in the main branch.^35^ Moreover, Doppler-derived velocity measurements at multiple locations throughout the artery were performed to obtain patient-specific boundary conditions for the CFD simulations. Previous WSS studies used a more simplified geometry and/or made more generalized assumptions about flow distribution.^29,34,36^ A broad between-subjects range in WSS was observed in our more detailed and more individually tailored model. Thus, vessel-specific WSS tertile has been selected in this study to identify the different WSS categories, which in an earlier study, proved to be most associated with plaque progression.^37^

### 4.3 Cross-sectional vs. sector-based analysis

The majority of the imaging studies identified lesions and plaques at the cross-sectional or segmental levels, reporting plaque burden, a normalized measure for plaque size. However, coronary atherosclerotic plaques are not equally distributed along the vessel and often have an eccentric shape.^38,39^ WSS plays a vital role in atheroprone sites, affecting plaque distribution along the coronary arteries. Most imaging studies do not take the spatial heterogeneity of WSS and its effect on plaque development into account. Timmins *et al*.^40^ showed that dividing the arteries, not only into cross-sectional segments but also into a more detailed sector-based analysis, significantly adds to the granularity of the data aiding in the focal evaluation of the natural history of coronary atherosclerosis. As a consequence of spatial averaging of the plaque and WSS data per cross-sectional segment, the local effects of WSS may be averaged out. In contrast, dividing segments into sectors gives greater insights into the plaque distribution and local WSS effects combined with the opportunity to investigate the WSS effect on a more homogenous type of plaque.

### 4.4 Limitations

In this study, several limitations need to be addressed. Firstly, we found an unexpectedly low number of sectors meeting the criteria of FCA (i.e. lipid pool); only 28 sectors were identified as per the guideline definitions.^11^ Therefore, all sectors containing lipids (both lipid pools and lipid-rich) have been pooled and analysed as one category. Statistical analysis on FCAs was not feasible due to the low numbers. In this study, the LDL levels were already found to be relatively low at the index procedure, and only 42% of the patients were statin-naïve at the time of enrolment, and this could have contributed to the low number of FCAs observed at baseline. Furthermore, in this study, no differences were found between patients with and without high-intensity lipid-lowering therapy. Only 50% of the patients remained adherent to high-intensity lipid-lowering therapy after a 1-year follow-up. Even though the cardiovascular benefits of high-intensity lipid-lowering therapy are known, low adherence is reported in daily clinical practice.^20^ In contrast to previous serial invasive imaging studies, designed as large lipid-lowering trials, our study was designed to investigate the natural history of atherosclerosis in non-culprit vessels of patients with an ACS in daily clinical practice and was not designed as a prospective comparison. Those lipid-lowering trials with a large number of patients showed minimal plaque regression for statin-naïve patients.^21,22^ With the relatively small number of patients investigated in this study, the absence of statistically significant differences in plaque progression of patients treated with high-intensity lipid-lowering therapy vs. low-to-moderate-intensity lipid-lowering therapy could be anticipated.

Secondly, by using multiple imaging modalities in this study, we still needed to co-register NIRS-IVUS and OCT pullbacks. Some minor errors could not be excluded despite our using as many side branches as possible when performing the co-registration. Therefore, the ROI was divided into 1.5 mm segments—on average, three IVUS frames per segment—to minimize the effect of longitudinal matching errors on the results. The advantage of the NIRS-IVUS-derived lipid data was that it was detected and exactly co-localized with the plaque volume data. Finally, the small number of patients did not allow us to draw any conclusions on the direct link between the interaction of WSS and LRPs and clinical events. Future studies or retrospective analyses of NIRS outcome studies may help understand this relationship in more detail.

## 5. Conclusion

In this study, we investigated the WSS-related plaque progression of LRPs compared with non-LRPs and a plaque-free wall. By using multimodality imaging techniques as input for our WSS analysis, co-localization of WSS and LRP and volumetric plaque assessment was possible. LRPs, as assessed by NIRS, showed greater plaque progression than non-LRPs. LRPs, identified by both NIRS and OCT, and exposed to low WSS, showed the highest plaque growth after a 1-year follow-up.

## Authors’ contributions

Conceptualization was done by E.M.J.H., A.M.K., F.J.H.G., and J.J.W. Data curation was done by E.M.J.H, A.M.K., M.T., F.M.A.N., W.K.d.D., R.D., N.M.D.A.v.M., J.D., J.J.W., and F.Z. Simulations were done by G.D.N., A.M.K., and S.-A.K. Post-processing of the results was done by E.M.J.H, G.D.N., A.M.K., and S.-A.K. Interpretation of data was done by E.M.J.H, J.D., and J.J.W. Supervision was done by J.D. and J.J.W. The first draft was prepared by E.M.J.H. All authors discussed the results and reviewed the manuscript.

## Data Availability

JW has access to the data that support the findings of this study. Restrictions apply to the availability of these data, which were collected and used under the METC approval of this study and the permission of the participants.

## References

[1] Mathers CD , Loncar D. Projections of global mortality and burden of disease from 2002 to 2030. PLoS Med 2006;3:e442. 17132052 10.1371/journal.pmed.0030442 PMC1664601

[2] Peiffer V , Sherwin SJ, Weinberg PD. Spotlight review: does low and oscillatory wall shear stress correlate spatially with early atherosclerosis? A systematic review. Cardiovasc Res 2013;99:242–250. 23459102 10.1093/cvr/cvt044 PMC3695746

[3] Chiu JJ , Chien S. Effects of disturbed flow on vascular endothelium: pathophysiological basis and clinical perspectives. Physiol Rev 2011;91:327–387. 21248169 10.1152/physrev.00047.2009 PMC3844671

[4] Chatzizisis YS , Coskun AU, Jonas M, Edelman ER, Feldman CL, Stone PH. Role of endothelial shear stress in the natural history of coronary atherosclerosis and vascular remodeling: molecular, cellular, and vascular behavior. J Am Coll Cardiol 2007;49:2379–2393. 17599600 10.1016/j.jacc.2007.02.059

[5] Libby P . Inflammation in atherosclerosis. Arterioscler Thromb Vasc Biol 2012;32:2045–2051. 22895665 10.1161/ATVBAHA.108.179705 PMC3422754

[6] Libby P , Hansson GK. From focal lipid storage to systemic inflammation: JACC review topic of the week. J Am Coll Cardiol 2019;74:1594–1607. 31537270 10.1016/j.jacc.2019.07.061 PMC6910128

[7] Choi B-J , Prasad A, Gulati R, Best PJ, Lennon RJ, Barsness GW, Lerman LO, Lerman A. Coronary endothelial dysfunction in patients with early coronary artery disease is associated with the increase in intravascular lipid core plaque. Eur Heart J. 2013;34:2047–2054. 23569198 10.1093/eurheartj/eht132 PMC3710580

[8] Ahmadi A , Leipsic J, Blankstein R, Taylor C, Hecht H, Stone GW, Narula J. Do plaques rapidly progress prior to myocardial infarction? The interplay between plaque vulnerability and progression. Circ Res 2015;117:99–104. 26089367 10.1161/CIRCRESAHA.117.305637

[9] Johnson TW , Räber L, Di Mario C, Bourantas C, Jia H, Mattesini A, Gonzalo N, De La Torre Hernandez JM, Prati F, Koskinas K, Joner M, Radu MD, Erlinge D, Regar E, Kunadian V, Maehara A, Byrne RA, Capodanno D, Akasaka T, Wijns W, Mintz GS, Guagliumi G. Clinical use of intracoronary imaging. Part 2: acute coronary syndromes, ambiguous coronary angiography findings, and guiding interventional decision-making: an expert consensus document of the European Association of Percutaneous Cardiovascular Interventions Interventional Cardiology. Eur Heart J 2019;40:1–19. 31112213 10.1093/eurheartj/ehz332

[10] Mintz GS , Maehara A. Serial intravascular ultrasound assessment of atherosclerosis progression and regression. State-of-the-art and limitations. Circ J 2009;73:1557–1560. 19638706 10.1253/circj.cj-09-0475

[11] Tearney GJ , Regar E, Akasaka T, Adriaenssens T, Barlis P, Bezerra HG, Bouma B, Bruining N, Cho JM, Chowdhary S, Costa MA, De Silva R, Dijkstra J, Di Mario C, Dudeck D, Falk E, Feldman MD, Fitzgerald P, Garcia H, Gonzalo N, Granada JF, Guagliumi G, Holm NR, Honda Y, Ikeno F, Kawasaki M, Kochman J, Koltowski L, Kubo T, Kume T, et al Consensus standards for acquisition, measurement, and reporting of intravascular optical coherence tomography studies: a report from the International Working Group for Intravascular Optical Coherence Tomography Standardization and Validation. J Am Coll Cardiol 2012;59:1058–1072. 22421299 10.1016/j.jacc.2011.09.079

[12] Roleder T , Kovacic JC, Ali Z, Sharma R, Cristea E, Moreno P, Sharma SK, Narula J, Kini AS. Combined NIRS and IVUS imaging detects vulnerable plaque using a single catheter system: a head-to-head comparison with OCT. EuroIntervention 2014;10:303–311. 24769522 10.4244/EIJV10I3A53

[13] Goldstein JA , Madden SP, Sum ST, Dixon SR, Madder RD, Muller JE. Assessment of plaque composition with near-infrared spectroscopy. Curr Cardiovasc Imaging Rep 2011;4:298–308.

[14] Schuurman A-S , Vroegindewey M, Kardys I, Oemrawsingh RM, Cheng JM, de Boer S, Garcia-Garcia HM, van Geuns R-J, Regar ES, Daemen J, van Mieghem NM, Serruys PW, Boersma E, Akkerhuis KM. Near-infrared spectroscopy-derived lipid core burden index predicts adverse cardiovascular outcome in patients with coronary artery disease during long-term follow-up. Eur Heart J 2018;39:295–302. 28531282 10.1093/eurheartj/ehx247

[15] Hartman EMJ , De Nisco G, Kok AM, Hoogendoorn A, Coenen A, Mastik F, Korteland S-A, Nieman K, Gijsen FJH, van der Steen AFW, Daemen J, Wentzel JJ. Lipid-rich plaques detected by near-infrared spectroscopy are more frequently exposed to high shear stress. J Cardiovasc Transl Res 2021;14:416–425. 33034862 10.1007/s12265-020-10072-x PMC8219563

[16] van der Giessen AG , Schaap M, Gijsen FJH, Groen HC, van Walsum T, Mollet NR, Dijkstra J, van de Vosse FN, Niessen WJ, de Feyter PJ, van der Steen AFW, Wentzel JJ. 3D Fusion of intravascular ultrasound and coronary computed tomography for in-vivo wall shear stress analysis: a feasibility study. Int J Cardiovasc Imaging 2010;26:781–796. 19946749 10.1007/s10554-009-9546-y

[17] De Nisco G , Kok AM, Chiastra C, Gallo D, Hoogendoorn A, Migliavacca F, Wentzel JJ, Morbiducci U. The atheroprotective nature of helical flow in coronary arteries. Ann Biomed Eng 2019;47:425–438. 30488307 10.1007/s10439-018-02169-x

[18] van der Giessen AG , Groen HC, Doriot P-A, de Feyter PJ, van der Steen AFW, van de Vosse FN, Wentzel JJ, Gijsen FJH. The influence of boundary conditions on wall shear stress distribution in patients specific coronary trees. J Biomech 2011;44:1089–1095. 21349523 10.1016/j.jbiomech.2011.01.036

[19] Mintz GS . Intravascular imaging of coronary calcification and its clinical implications. JACC Cardiovasc Imaging 2015;8:461–471. 25882575 10.1016/j.jcmg.2015.02.003

[20] Vonbank A , Drexel H, Agewall S, Lewis BS, Dopheide JF, Kjeldsen K, Ceconi C, Savarese G, Rosano G, Wassmann S, Niessner A, Schmidt TA, Saely CH, Baumgartner I, Tamargo J. Reasons for disparity in statin adherence rates between clinical trials and real-world observations: a review. Eur Heart J Cardiovasc Pharmacother 2018;4:230–236. 30099530 10.1093/ehjcvp/pvy028

[21] Nissen SE , Nicholls SJ, Sipahi I, Libby P, Raichlen JS, Ballantyne CM, Davignon J, Erbel R, Fruchart JC, Tardif JC, Schoenhagen P, Crowe T, Cain V, Wolski K, Goormastic M, Tuzcu EM. Effect of very high-intensity statin therapy on regression of coronary atherosclerosis: the ASTEROID trial. J Am Med Assoc 2006;295:1556–1565. 10.1001/jama.295.13.jpc60002 16533939

[22] Oemrawsingh RM , Garcia-Garcia HM, Van Geuns RJM, Lenzen MJ, Simsek C, De Boer SPM, Van Mieghem NM, Regar E, De Jaegere PPT, Akkerhuis KM, Ligthart JMR, Zijlstra F, Serruys PW, Boersma E. Integrated Biomarker and Imaging Study 3 (IBIS-3) to assess the ability of rosuvastatin to decrease necrotic core in coronary arteries. EuroIntervention 2016;12:734–739. 27542785 10.4244/EIJV12I6A118

[23] Patel DD , Hamamdzic D, Llano R, Patel DD, Cheng L, Fenning RS, Bannan K, Wilensky RL. Subsequent development of fibroatheromas with inflamed fibrous caps can be predicted by intracoronary near infrared spectroscopy. Arterioscler Thromb Vasc Biol 2013;33:347–353. 23288155 10.1161/ATVBAHA.112.300710

[24] Waksman R , Di Mario C, Torguson R, Ali ZA, Singh V, Skinner WH, Artis AK, Ten Cate T, Powers E, Kim C, Regar E, Wong SC, Lewis S, Wykrzykowska J, Dube S, Kazziha S, van der Ent M, Shah P, Craig PE, Zou Q, Kolm P, Brewer HB, Garcia-Garcia HM, Samady H, Tobis J, Zainea M, Leimbach W, Lee D, Lalonde T, Skinner W, et al Identification of patients and plaques vulnerable to future coronary events with near-infrared spectroscopy intravascular ultrasound imaging: a prospective, cohort study. Lancet 2019;394:1629–1637. 31570255 10.1016/S0140-6736(19)31794-5

[25] Gardner CM , Tan H, Hull EL, Lisauskas JB, Sum ST, Meese TM, Jiang C, Madden SP, Caplan JD, Burke AP, Virmani R, Goldstein J, Muller JE. Detection of lipid core coronary plaques in autopsy specimens with a novel catheter-based near-infrared spectroscopy system. JACC Cardiovasc Imaging 2008;1:638–648. 19356494 10.1016/j.jcmg.2008.06.001

[26] Di Vito L , Imola F, Gatto L, Romagnoli E, Limbruno U, Marco V, Picchi A, Micari A, Albertucci M, Prati F. Limitations of OCT in identifying and quantifying lipid components: an in vivo comparison study with IVUS-NIRS. EuroIntervention 2017;13:303–311. 27973332 10.4244/EIJ-D-16-00317

[27] Malek AM , Alper SL, Izumo S. Hemodynamic shear stress and its role in atherosclerosis. JAMA 1999;282:2035–2042. 10591386 10.1001/jama.282.21.2035

[28] Samady H , Eshtehardi P, McDaniel MC, Suo J, Dhawan SS, Maynard C, Timmins LH, Quyyumi AA, Giddens DP. Coronary artery wall shear stress is associated with progression and transformation of atherosclerotic plaque and arterial remodeling in patients with coronary artery disease. Circulation 2011;124:779–788. 21788584 10.1161/CIRCULATIONAHA.111.021824

[29] Stone PH , Saito S, Takahashi S, Makita Y, Nakamura SS, Kawasaki T, Takahashi A, Katsuki T, Nakamura SS, Namiki A, Hirohata A, Matsumura T, Yamazaki S, Yokoi H, Tanaka S, Otsuji S, Yoshimachi F, Honye J, Harwood D, Reitman M, Coskun AU, Papafaklis MI, Feldman CL, PREDICTION Investigators. Prediction of progression of coronary artery disease and clinical outcomes using vascular profiling of endothelial shear stress and arterial plaque characteristics: the PREDICTION study. Circulation 2012;126:172–181. 22723305 10.1161/CIRCULATIONAHA.112.096438

[30] Shishikura D , Sidharta SL, Honda S, Takata K, Kim SW, Andrews J, Montarello N, Delacroix S, Baillie T, Worthley MI, Psaltis PJ, Nicholls SJ. The relationship between segmental wall shear stress and lipid core plaque derived from near-infrared spectroscopy. Atherosclerosis 2018;275:68–73. 29864607 10.1016/j.atherosclerosis.2018.04.022

[31] Slager C , Wentzel J, Gijsen F, Thury A, van der Wal A, Schaar J, Serruys P. The role of shear stress in the destabilization of vulnerable plaques and related therapeutic implications. Nat Clin Pract Cardiovasc Med 2005;2:456–464. 16265586 10.1038/ncpcardio0298

[32] Stone PH , Maehara A, Coskun AU, Maynard CC, Zaromytidou M, Siasos G, Andreou I, Fotiadis D, Stefanou K, Papafaklis M, Michalis L, Lansky AJ, Mintz GS, Serruys PW, Feldman CL, Stone GW. Role of low endothelial shear stress and plaque characteristics in the prediction of nonculprit major adverse cardiac events: the PROSPECT study. JACC Cardiovasc Imaging 2018;11:462–471. 28917684 10.1016/j.jcmg.2017.01.031

[33] Bourantas CV , Räber L, Sakellarios A, Ueki Y, Zanchin T, Koskinas KC, Yamaji K, Taniwaki M, Heg D, Radu MD, Papafaklis MI, Kalatzis F, Naka KK, Fotiadis DI, Mathur A, Serruys PW, Michalis LK, Garcia-Garcia HM, Karagiannis A, Windecker S. Utility of multimodality intravascular imaging and the local hemodynamic forces to predict atherosclerotic disease progression. JACC Cardiovasc Imaging 2020;13:1021–1032. 31202749 10.1016/j.jcmg.2019.02.026

[34] Bourantas CV , Zanchin T, Sakellarios A, Karagiannis A, Ramasamy A, Yamaji K, Taniwaki M, Heg D, Moschovitis A, Fotiadis D, Mihalis L, Baumbach A, Torii R, Serruys P, Garcia-Garcia HM, Windecker S, Räber L. Implications of the local haemodynamic forces on the phenotype of coronary plaques. Heart 2019;105:1078–1086. 30877239 10.1136/heartjnl-2018-314086

[35] Li Y , Gutiérrez-Chico JL, Holm NR, Yang W, Hebsgaard L, Christiansen EH, Mæng M, Lassen JF, Yan F, Reiber JHC, Tu S. Impact of side branch modeling on computation of endothelial shear stress in coronary artery disease. J Am Coll Cardiol 2015;66:125–135. 26160628 10.1016/j.jacc.2015.05.008

[36] Yamamoto E , Siasos G, Zaromytidou M, Coskun AU, Xing L, Bryniarski K, Zanchin T, Sugiyama T, Lee H, Stone PH, Jang I-K. Low endothelial shear stress predicts evolution to high-risk coronary plaque phenotype in the future. Circ Cardiovasc Interv 2017;10:e005455. 28768758 10.1161/CIRCINTERVENTIONS.117.005455

[37] Hartman EMJ , De Nisco G, Gijsen FJH, Korteland S-A, van der Steen AFW, Daemen J, Wentzel JJ. The definition of low wall shear stress and its effect on plaque progression estimation in human coronary arteries. Sci Rep 2021;11:22086. 34764316 10.1038/s41598-021-01232-3 PMC8586146

[38] Caro GG . Discovery of the role of wall shear in atherosclerosis. Arterioscler Thromb Vasc Biol 2009;29:158–161. 19038849 10.1161/ATVBAHA.108.166736

[39] Fox B , James K, Morgan B, Seed A. Distribution of fatty and fibrous plaques in young human coronary arteries. Atherosclerosis 1982;41:337–347. 7066081 10.1016/0021-9150(82)90198-8

[40] Timmins LH , Molony DS, Eshtehardi P, McDaniel MC, Oshinski JN, Samady H, Giddens DP. Focal association between wall shear stress and clinical coronary artery disease progression. Ann Biomed Eng 2014;43:94–106. 25316593 10.1007/s10439-014-1155-9

